# Case Report: Plummer's adenoma in Prader–Willi syndrome

**DOI:** 10.3389/fped.2024.1388437

**Published:** 2024-08-08

**Authors:** Domenico Corica, Fabio Toscano, Mariacarla Moleti, Giorgia Pepe, Alfredo Campenni, Guido Fadda, Gianlorenzo Dionigi, Carmelo Romeo, Tommaso Aversa, Malgorzata Wasniewska

**Affiliations:** ^1^ Pediatric Unit, Department of Human Pathology of Adulthood and Childhood “G. Barresi”, University of Messina, Messina, Italy; ^2^ Endocrinology Unit, Department of Clinical and Experimental Medicine, University of Messina, Messina, Italy; ^3^ Nuclear Medicine Unit, Department of Biomedical and Dental Sciences and Morpho-Functional Imaging, University of Messina, Messina, Italy; ^4^ Section of Pathological Anatomy, Department of Human Pathology of Adulthood and Childhood “Gaetano Barresi”, University of Messina, Messina, Italy; ^5^ Division of Surgery, Istituto Auxologico Italiano, Istituto di Ricovero e Cura a Carattere Scientifico (IRCCS), Milano, Italy; ^6^ Pediatric Surgery Unit, Department of Human Pathology of Adulthood and Childhood “Gaetano Barresi”, University of Messina, Messina, Italy

**Keywords:** Plummer’s adenoma, subclinical hyperthyroidism, children, thyroid nodule, GH therapy

## Abstract

Thyroid nodules in children are less common than in adults but they are approximately two- to three-fold more likely to be malignant in children. Among thyroid nodular diseases, Plummer's adenoma occurs very rarely in pediatrics, and currently, there is no literature providing evidence of this diagnosis in patients with Prader–Willi syndrome (PWS). We report the case of a 9-year-old Caucasian boy affected by PWS presenting with a rapidly growing palpable mass in the thyroid lodge associated with subclinical hyperthyroidism. Laboratory and other examinations (thyroid ultrasound, fine-needle aspiration of the nodule, and scintigraphy) were strongly suggestive for Plummer's adenoma; therefore, the patient underwent left hemithyroidectomy surgery, and anatomo-pathological examination confirmed the diagnosis. Our case describes the first evidence of an isolated follicular adenoma in children with PWS. Surgery is the only therapeutic option in younger children. Further evidence is needed to assess the possible correlation between these two conditions and the existence of potential risk factors.

## Introduction

Thyroid nodular disease is very rare at pediatric ages. Approximately 2% of children and adolescents have palpable thyroid nodules and 0.5%–5% have ultrasonography-demonstrated nodules in the gland (1–3). Most thyroid nodules in children are benign but approximately 26% represent a differentiated thyroid cancer (DTC); therefore, although thyroid nodules in children are less common than in adults, they are approximately two- to three-fold more likely to be malignant (4). Children with thyroid nodules should be evaluated with serum thyroid-stimulating hormone (TSH) and free thyroxine (fT4), and thyroid ultrasound. Usually, the majority of children are euthyroid; in this case, a fine-needle aspiration (FNA) of the nodule should be performed if it is >1 cm or, regardless of the size, there is a suspicious clinical context or there are specific ultrasound features of malignancy (5). Furthermore, an increase in nodule size during the follow-up is considered an index of malignancy, and it is appropriate to repeat an FNA when the previous sampling was non-diagnostic (6). In 5% of cases, thyroid nodules occur with hyperthyroidism; in the presence of low/suppressed TSH levels, a Plummer's adenoma (PA) is suspected and a thyroid scintigraphy should be performed (5).

PA (or toxic adenoma or autonomous functioning thyroid adenoma) accounts for approximately 5%–10% of all solitary nodules and occurs predominantly in females (13:1), more commonly in older age groups (7). In children and adolescents, PA is an unusual finding but it seems to have a more rapidly progressive course than those in adults (8). Increased radioiodine uptake within the nodule on thyroid scintigraphy, associated with a suppression in the surrounding tissue of the gland, is consistent with PA (5). These lesions are most frequently associated with somatic activating mutations within the genes encoding the TSH receptor or the Gs-alpha subunit (9). To date, up to one-third of patients may have an incidentally discovered DTC associated with autonomous nodules. In this regard, surgical treatment (lobectomy plus isthmusectomy in most cases) is the recommended approach for most pediatric patients and patients below 10 years of age, considering that below that age, I-131 ablation is regarded as a contraindication according to the most recent European Association of Nuclear Medicine (EANM) guidelines (10), and it should be proposed at the time of the diagnosis (5).

Prader–Willi Syndrome (PWS), the most common cause of genetic obesity in the pediatric population, is a complex disorder with hypothalamus-pituitary axis abnormalities (anterior pituitary hypoplasia and an absent, small, or ectopic posterior pituitary gland) in more than 50% of patients, possibly resulting in endocrinological dysfunctions [growth hormone (GH) deficiency, hypogonadotropic hypogonadism, adrenal insufficiency, and hypothyroidism] (11–15). In this regard, the most common thyroid dysfunction in PWS seems to be central hypothyroidism (CH) (16, 17), the incidence of which varies from 2% to 32% (12, 14, 18, 19). However, some authors have reported a prevalence of CH in PWS that is similar to the general population (20, 21). Rare cases of congenital hypothyroidism (22, 23) and fetal goiter (24) have been reported, whereas descriptions of hyperthyroidism cases are sporadic (25). To date, no PA cases in either adult or pediatric subjects with PWS have been described in the scientific literature. In this report, we describe the first case of PA reported in a child with PWS presenting as a rapidly growing thyroid nodular lesion and subclinical hyperthyroidism.

## Case report

A 9-year-old boy, followed at our Pediatric Endocrinology Outpatients Clinic for PWS, owing to maternal uniparental disomy for chromosome 15, presented with the appearance of a palpable non-painful mass at the left anterior cervical site in the thyroid lodge. The patient, who was receiving recombinant human growth hormone (rhGH) therapy since the age of 1 year, had regular growth in height and weight; at the time of the examination he had a stature of 130 cm (+0.05 SD), a weight of 36.7 kg (+1.48 SD), a body mass index of 21.75 kg/m^2^ (+1.67 SD), and a regular growth rate (7.6 cm/year). The child was prepubescent in accordance with the Tanner Stage. Familial history was negative for thyroid diseases. During outpatient follow-up, thyroid function was always within normal limits and anti-thyroid antibodies were negative, as was the case at the last check-up 6 months before the diagnosis of the palpable neck mass: TSH, 1.2 uIU/ml [normal value (n.v.) 0.27–4.2]; free triiodothyronine (fT3), 3.6 pg/ml (n.v. 2.0–4.4); and fT4, 19 pmol/L (n.v. 12.0–22.0); anti-thyroglobulin (AbTg), anti-peroxidase (AbTPO), and TSH receptor (TRAb) antibodies were negative.

**Figure 1:**
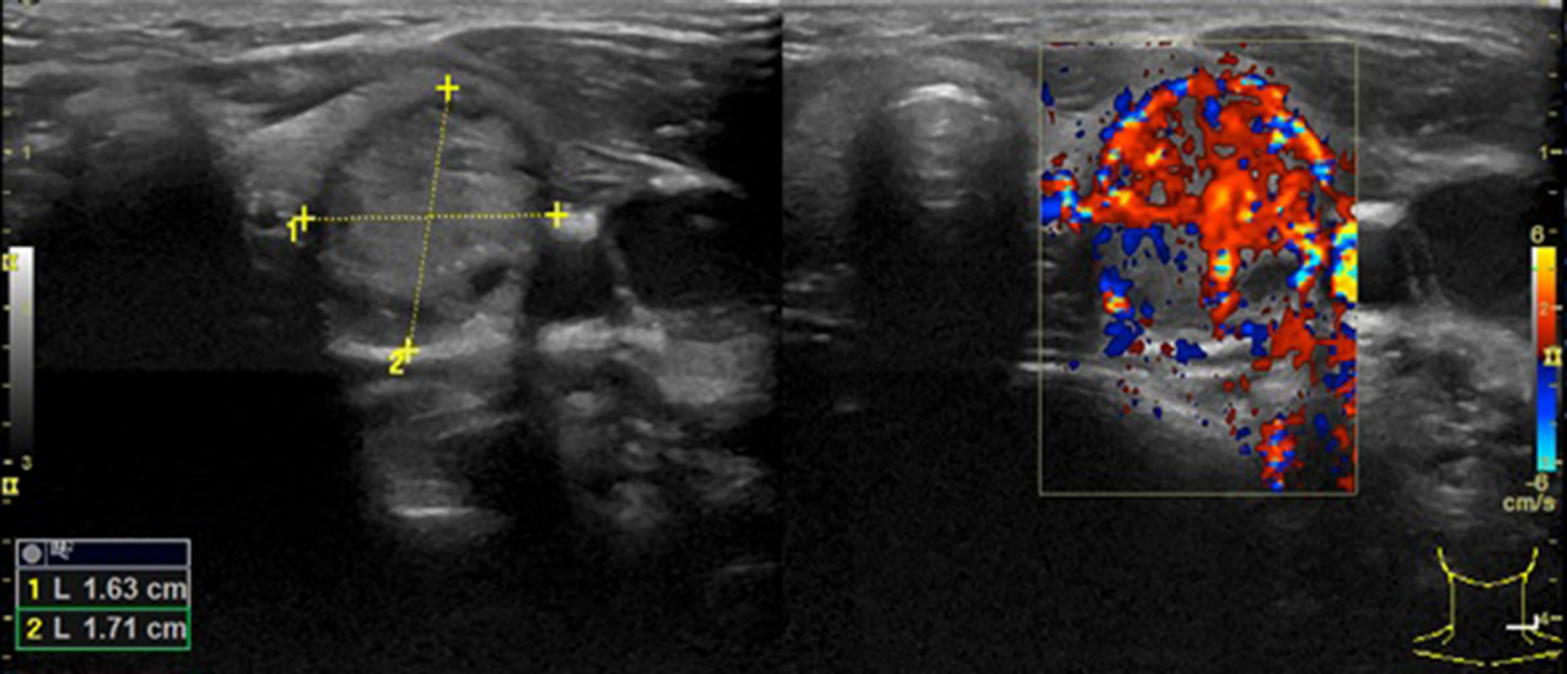
The first thyroid ultrasound examination showing a solitary thyroid nodule with an LD of 17 mm in the left lobe.

**Figure 2:**
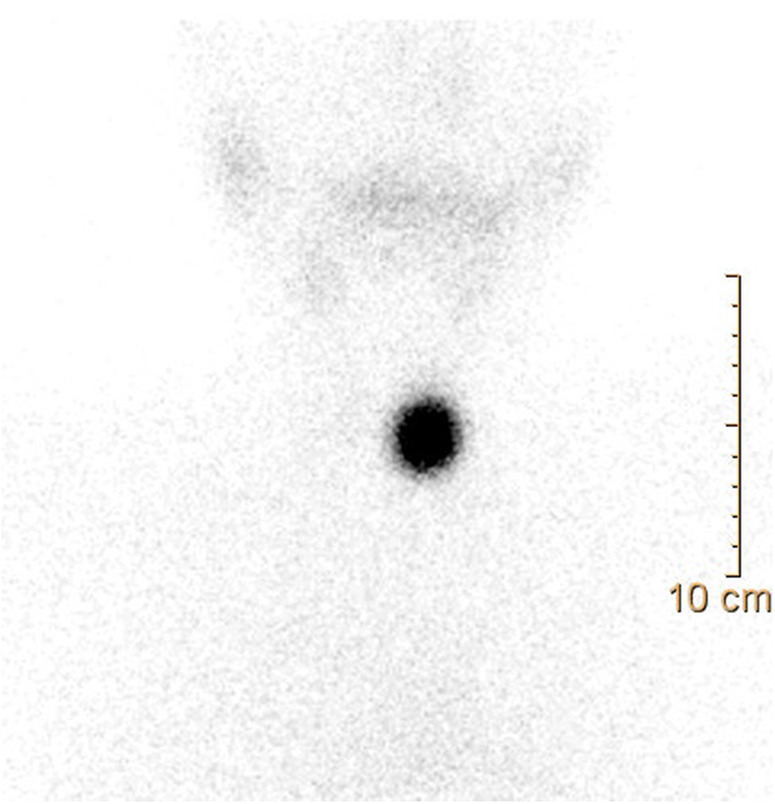
Thyroid scintigraphy: a scintigraphic exam documenting a hyperfunctioning nodule, as shown by the focal iodine-131 concentration on the nodule with suppression of the surrounding thyroid gland tissue.

**Figure 3:**
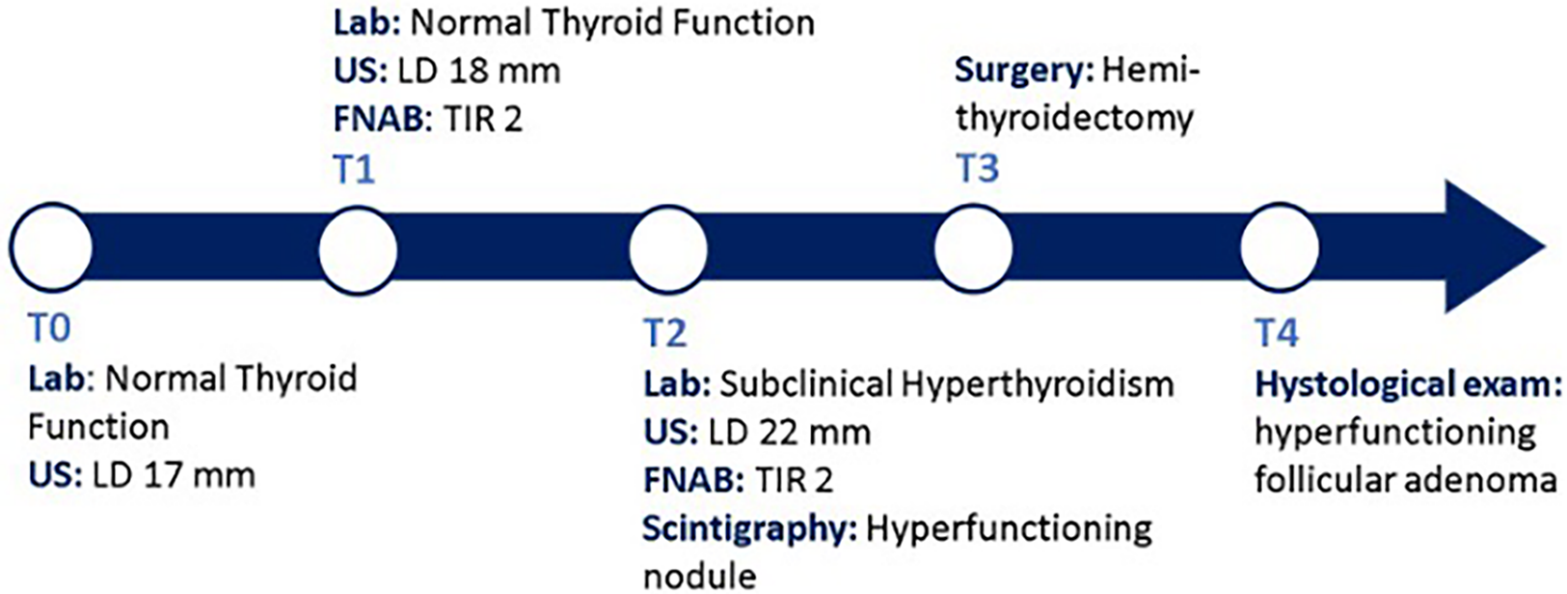
Diagnostic and therapeutic timeline. **T0**, the patient came to our attention with a palpable thyroid mass with an LD of 17 mm at the US exam, within normal thyroid function. **T1**, A FNAB was conducted due to the ultrasound dimensions of the nodule; malign causes were excluded. **T2**, after 6 months, the nodule grew up to 22 mm in LD, with evidence of some risk factors at the US exam, and TSH suppression was documented; a second FNAB confirmed a non-malignant lesion (TIR2) and the thyroid scintigraphy documented a focal iodine-132 concentration on the nodule with suppression of the surrounding gland tissue. **T3**, the patient underwent hemithyroidectomy surgery. **T4**, the histological examination confirmed the diagnosis of PA. Lab, laboratory exams; US, ultrasound; FNAB, fine-needle aspiration biopsy; PA, Plummer's adenoma; TSH, thyroid-stimulating hormone.

Following the aforementioned finding, the child therefore underwent a thyroid ultrasound that documented the presence of a nodule in the left lobe [longitudinal diameter (LD), 17 mm] with a disomogeneous structure and microcalcifications, and without pathological findings at the color-Doppler evaluation (shown in Figure 1). Thus, considering nodule dimension, in accordance with the most recent guidelines (5), the patient underwent a fine-needle aspiration biopsy (FNAB), which suggested a non-malignant lesion (TIR2 according to the 2014 Italian consensus for the classification of thyroid cytology) (26). Over approximately 6 months, thyroid biochemical assessment shifted toward a subclinical hyperthyroidism: TSH, 0.005 uIU/ml; fT4, 20.4 pmol/L; and fT3 5.79 pg/ml. Thyroid autoantibodies were still negative. At the ultrasound reassessment, the nodule had grown in size (LD, 22 mm) and increased vascular signals were evident. A second FNAB confirmed the presence of a non-malignant lesion/adenomatous struma (TIR2) (26). Owing to the TSH suppression, a scintigraphic examination was carried out, which documented a defined focal uptake of iodine-123 in the nodule with suppressed uptake in the rest of the gland (shown in Figure 2). In light of the rapid and progressive increase in nodule size, the biochemical pattern of subclinical hyperthyroidism, and the scintigraphic finding of a hyper capturing nodule, the patient underwent left hemithyroidectomy surgery. The anatomo-pathological examination revealed a follicular adenoma, confirming the diagnosis of PA, that was hyperfunctioning on the basis of the hormonal profile. Figure 3 shows the timeline from the first clinical finding of the thyroid nodule to the diagnosis of PA. Two months after the surgery, the boy developed an acquired hypothyroidism, possibly due to a limited functionality of the residual thyroid tissue. Levothyroxine therapy was started. Five months after the hemithyroidectomy and the start of levothyroxine therapy, euthyroidism was maintained with a 1 μg/kg/day dose of levothyroxine.

## Discussion

The clinical case described in this report is the first evidence of PA in a child with PWS. PA seems to be extremely rare in children and adolescents, even when referring to the general population. There are limited data about the incidence of PA in children. Toxic adenoma has been reported in 5%–7.5% of pediatric patients with thyroid nodules (27). Children with a hyperfunctioning autonomous nodule may indeed present with either mild or overt hyperthyroidism or sometimes euthyroidism (5). There seems to be a correlation between the size of the nodule and the biochemical picture. It has been reported that autonomous nodules less than 2.5 cm in diameter usually show incomplete suppression of the surrounding thyroid parenchyma, resulting in a subclinical hyperthyroidism. On the other hand, complete suppression is usually associated with lesions of 3 cm or more in size (7). However, the natural history of this rare condition and the correlation between the biochemical and radiological picture of the (complete or incomplete) suppression of the thyroid parenchyma surrounding the lesion have not been well defined (5). The available data on the prevalence and evolution of thyroid diseases in subjects with PWS almost exclusively concern CH and congenital hypothyroidism (14, 19), thus there are insufficient data to hypothesize a peculiar evolution of the thyroid biochemical picture of PA in PWS.

The most recent guidelines recommend surgery, lobectomy plus isthmectomy in most cases, as the optimal initial management for children with PA because of concerns about the mutagenic effect of radioiodine on the normal thyroid tissue, supported by the concern that the cancer risk in children with a thyroid nodular disease is higher than in the adult population and up to one-third of patients may have an incidentally discovered cancer associated with autonomous nodules (5, 7).

PWS is not considered a cancer predisposition syndrome: a recent retrospective study with a large international cohort of 706 patients confirmed that malignancies are rare in patients with PWS and usually due to a multifactorial etiology (28). To date, 50 published reports describe malignancies in individuals with PWS (29), including acute lymphoblastic leukemia (30), acute and chronic myeloid leukemia (31), hepatoblastoma (32), medulloblastoma (33), pulmonary carcinoid tumor (34), Wilms tumor (35), multiple endocrine neoplasia type 1 (36), a case of fetal cardiac rhabdomyoma (37), and gonadal germ cell tumors in 22.5% of all cases (11/50) (29). Maya-González et al. showed no increased risk of cancer at any age in PWS patients compared with the general population; however, among individuals developing cancer, they highlighted an increased prevalence of tumors at pediatric age in PWS patients compared with the control group (25% vs. 8.7%) (29). Moreover, they noticed an increased occurrence of gonadal (testicular or ovarian) tumors in PWS at any age in comparison with the control group (17% vs. 3%), suggesting an increased risk for these neoplasms in PWS (29). Furthermore, these authors (29) suggested a loss of imprinting (LOI) as a possible mechanism of tumorigenesis in these patients, as documented in their case report (29) and in a 13-year-old girl with PWS who also developed bilateral ovarian sex cord tumors with annular tubules (38).

The role of GH therapy in influencing the risk of cancer in PWS has been debated. GH treatment has shown significant beneficial effects on linear growth, body composition, physical strength, and mental development, with a reassuring safety profile for daily administration in children with PWS (39–41). Sjöström and Höybye documented that, over a follow-up period of approximately 20 years, GH treatment during childhood or in adulthood with PWS is not associated with an increased risk of cancer (42). Hirsch and Gross-Tsur hypothesized that increases in insulin-like factor-1 as a result of GH treatment over the course of several decades in PWS adults raise concern over the possible increase in the risk of cancer (43), although there is no evidence to date of a clear correlation between GH therapy and cancer risk in PWS subjects (14, 28). Multiple observational studies carried out in a non-PWS population did not indicate an increased risk of malignancy after treatment with GH during childhood (44, 45). However, it should be considered that the *Safety and Appropriateness of Growth Hormone Treatments in Europe* (SAGhE) study showed an increased incidence of cancer related to second primary malignancies in patients who received GH treatment after cancer treatment (46). Therefore, GH treatment is considered safe, although some authors suggest that the involvement of additional genetic factors and/or GH treatment in cancer development cannot be excluded (28, 30, 46).

In the few studies available, the malignancy rate of hyperfunctioning nodules at pediatric age ranges from 0% to 29% (27): Niedziela et al. reported an incidence of 29% malignancy among 31 children and adolescents with hyperfunctioning nodules detected by 99mTc scintigraphy (47). A group in Belo Horizonte, Brazil, reported an incidence of malignancy of 5.9% in a series of 13 patients with 17 hyperfunctioning nodules (48). On the other hand, Ly et al. reported no cancer in their single-center experience with 31 children diagnosed with PA, suggesting that conservative management could be offered in childhood, deferring definitive therapies until adulthood (49). Gundgurthi et al. reported the case of a 3-year-old boy with PA successfully treated with 131-iodine radio ablation (50).

Concerning our patient, no noticeable risk factors for cancer, such as obesity, a previous primary malignancy before starting GH treatment, or a suspected genetic predisposition, have been revealed according to the underlying syndrome. Nevertheless, in accordance with the most recent guidelines (5, 7), taking into account case reports of DTC found after the resection of a PA that support hemithyroidectomy as the treatment of choice (51–53), and considering that our patient with PWS and PA is a male younger than 10 years of age, with an unknown risk of malignancy in nodular disease, we decided to proceed with a hemithyroidectomy.

## Conclusions

To the best of our knowledge, this is the first report describing PA in a patient with PWS. The management of these rare cases should refer to the most recent guidelines in the management of pediatric thyroid pathology, taking into account the patient's characteristics. Further evidence is needed to assess a possible correlation between PWS and PA, the existence of potential risk factors associated with the appearance of these lesions, and the potential risk of malignancy of hyperfunctioning nodules in PWS.

## Data Availability

The original contributions presented in the study are included in the article/Supplementary Material, further inquiries can be directed to the corresponding author.
